# CX3CR1 Deficiency Attenuates DNFB-Induced Contact Hypersensitivity through Skewed Polarization towards M2 Phenotype in Macrophages

**DOI:** 10.3390/ijms21197401

**Published:** 2020-10-07

**Authors:** Sayaka Otobe, Teruyoshi Hisamoto, Tomomitsu Miyagaki, Sohshi Morimura, Hiraku Suga, Makoto Sugaya, Shinichi Sato

**Affiliations:** 1 Department of Dermatology, the University of Tokyo Graduate School of Medicine, Tokyo 113-8655, Japan; confiture.sayaka.95@gmail.com (S.O.); teru.hisamoto@gmail.com (T.H.); siccommunication@yahoo.co.jp (S.M.); sugah-der@h.u-tokyo.ac.jp (H.S.); sugayamder@gmail.com (M.S.); satos-der@h.u-tokyo.ac.jp (S.S.); 2 Department of Dermatology, St. Marianna University School of Medicine, Kanagawa 216-8511, Japan; 3 Department of Dermatology, International University of Health and Welfare, Chiba 286-0124, Japan

**Keywords:** CX3CR1, contact hypersensitivity, macrophage, tumor necrosis factor-α, interleukin-6, arginase-1

## Abstract

CX3CL1 can function as both an adhesion molecule and a chemokine for CX3CR1^+^ cells, such as T cells, monocytes, and NK cells. Recent studies have demonstrated that CX3CL1–CX3CR1 interaction is associated with the development of various inflammatory skin diseases. In this study, we examined CX3CR1 involvement in 2,4-dinitrofluorobenzene (DNFB)-induced contact hypersensitivity using CX3CR1^−/−^ mice. Ear swelling and dermal edema were attenuated after DNFB challenge in CX3CR1^−/−^ mice. Expression of TNF-α, IL-6, and M1 macrophage markers was decreased in the ears of CX3CR1^−/−^ mice, whereas expression of M2 macrophage markers including arginase-1 was increased. Decreased TNF-α and IL-6 expression and increased arginase-1 expression were found in peritoneal macrophages from CX3CR1^−/−^ mice. Furthermore, ear swelling was attenuated by depleting dermal macrophages in wild-type mice to a similar level to CX3CR1^−/−^ mice. These results suggest that CX3CR1 deficiency could induce skewed polarization towards M2 phenotype in macrophages, resulting in attenuation of contact hypersensitivity response.

## 1. Introduction

Contact hypersensitivity (CHS) is a cutaneous immunological response against a small chemical hapten including 2,4-dinitrofluorobenzene (DNFB), fluorescein isothiocyanate, and oxazolone. Clinically, various allergens such as metals, plants, and drugs can cause allergic contact dermatitis [1,2]. CHS involves two phases: the sensitization phase and the elicitation phase [3]. In the sensitization phase, activated dendritic cells and Langerhans cells, which take up the hapten, migrate to the legional lymph nodes, and present the hapten to naïve T cells, resulting in the formation of hapten-specific memory T cells [4]. In the elicitation phase, effector memory T cell activation and recruitment to the site are induced by re-exposure to the same hapten. T cells cause injury to the skin via production of inflammatory cytokines and activation of other immune cell populations, such as NK cells and macrophages. In this context, chemokines and chemokine receptors are deeply involved in the process of CHS via trafficking of antigen-presenting cells to the lymph node and recruiting of inflammatory cells to the hapten-applied site [5]. Moreover, chemokine receptor expression on immune cells can also affect the inflammatory characters of the cells. For example, CXCR3-deficient regulatory T cells have less capacity to produce suppressive cytokines, such as interleukin (IL)-10 and transforming growth factor-β; accordingly, DNFB-induced CHS was prolonged in CXCR3^−/−^ mice [6].

CX3CL1, a CX3C chemokine, is expressed by inflamed endothelial cells, vascular smooth muscle cells, and epithelial cells, and its receptor CX3CR1 is detected on T cells, monocytes, and NK cells [7]. CX3CL1 exists in two forms, a membrane-bound form and a soluble form, mediating distinct biologic actions, respectively. In the membrane-bound form, CX3CL1 functions as an adhesion molecule for CX3CR1-positive cells [8], while in the soluble form, it plays a key role in the migration of monocytes, NK cells, and T cells [9,10]. Recent studies have demonstrated that the CX3CL1–CX3CR1 interaction is associated with the development of various inflammatory diseases such as rheumatoid arthritis, inflammatory bowel diseases, and inflammatory skin disease [11,12]. Serum CX3CL1 levels were increased and CX3CR1^+^ cell infiltration was found in the lesional skin from atopic dermatitis (AD) patients [13,14]. In an AD model induced by epicutaneous sensitization with *Leishmania major*-activated C kinase, CX3CR1 expressed by CD4^+^ T cells contributed to their retention into the inflamed skin and exacerbation of dermatitis [14]. These results suggest that CX3CR1 can be involved in the development of AD. Furthermore, it was recently reported that attenuation of imiquimod-induced psoriasis-like skin inflammation was shown in CX3CR1^−/−^ mice through decrease in M1 macrophages [15]. Two single nucleotide polymorphisms in the CX3CR1 gene were associated with psoriasis [16] and CX3CR1 messenger RNA (mRNA) expression was upregulated in the lesional skin of psoriasis patients [17], suggesting that CX3CR1 can also be involved in the development of psoriasis. On the other hand, little is known about the contribution of CX3CR1 to the development of CHS. In this study, we examined the role of CX3CR1 in DNFB-induced CHS using CX3CR1^−/−^ mice.

## 2. Results

### 2.1. CX3CR1 Deficiency Attenuates CHS Response Induced by DNFB

**Figure 1:**
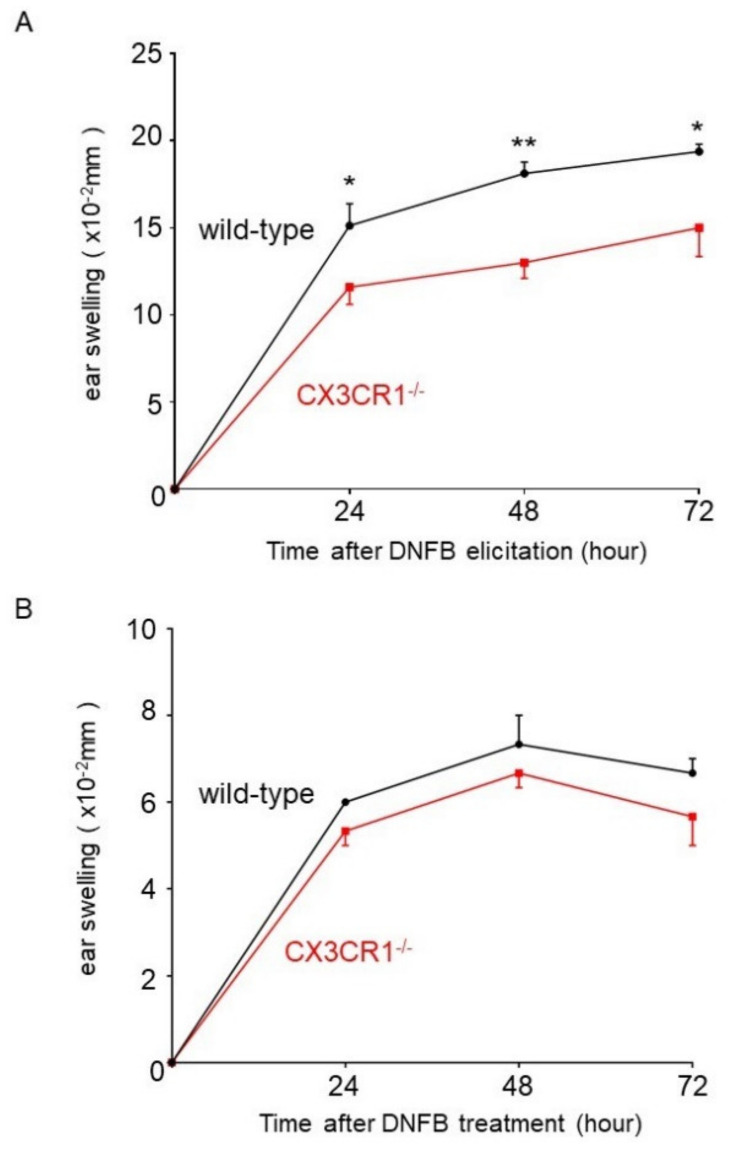
DNFB (2,4-dinitrofluorobenzene)-induced CHS (contact hypersensitivity) is attenuated in CX3CR1^−/−^ mice. (**A**) Wild-type mice and CX3CR1^−/−^ mice were sensitized with 0.5% DNFB on days 0 and 1. CHS was elicited by 0.5% DNFB on the right ear on day 6. Ear swelling was measured before elicitation and at 24, 48, and 72 h after elicitation and the changes in ear thickness from baseline measurement were presented. All values represent the mean ± SEM. *n* = 8–9. * *p* < 0.05, ** *p* < 0.01 by Mann–Whitney U-test. (**B**) Wild-type mice and CX3CR1^−/−^ mice were treated with 0.5% DNFB on the right ear without sensitization. Ear swelling was measured before and at 24, 48, and 72 h after treatment and the changes in ear thickness from baseline measurement were presented. All values represent the mean ± SEM. *n* = 4.

To assess the roles of CX3CR1 in CHS, CX3CR1^−/−^ and wild-type mice were challenged with DNFB after sensitization, and ear swelling was measured before and after the challenge. Ear swelling was significantly diminished in CX3CR1^−/−^ mice compared with wild-type mice 24, 48, and 72 h after elicitation (Figure 1A). On the other hand, ear swelling after DNFB challenge without sensitization in CX3CR1^−/−^ mice was similar to that in wild-type mice (Figure 1B). Thus, CX3CR1 deficiency attenuated CHS response induced by DNFB.

### 2.2. Dermal Edema and Infiltration of Neutrophils Were Decreased in CX3CR1^−/−^ Mice

**Figure 2:**
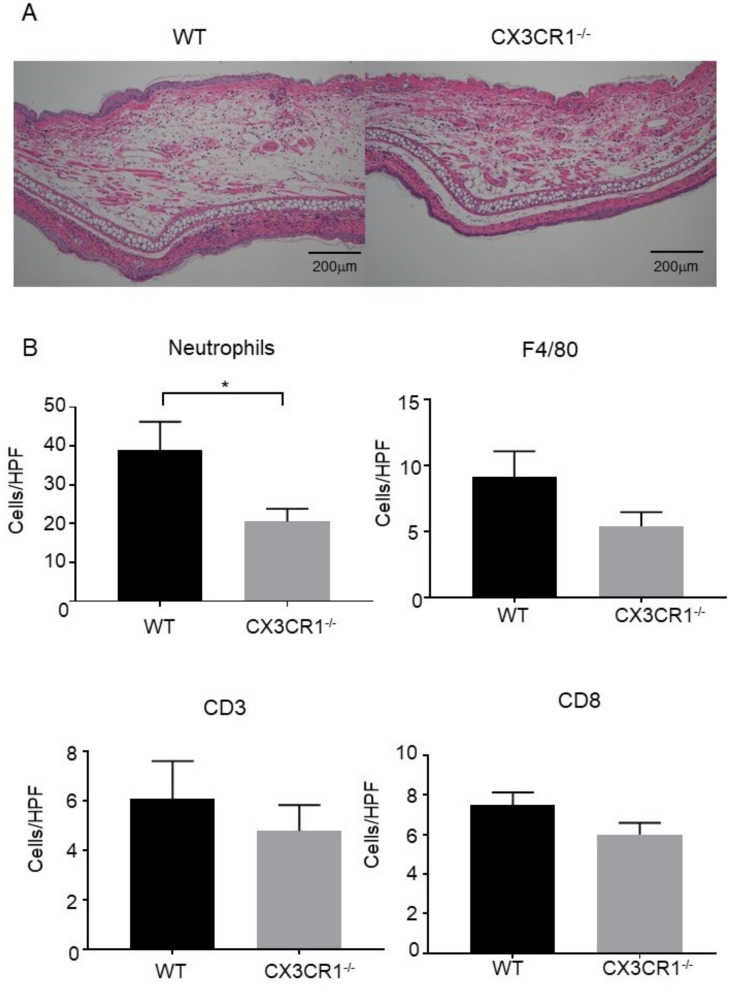
Dermal edema and neutrophil infiltration are decreased in CX3CR1^−/−^ mice. (**A**) Hematoxylin and eosin staining of skin samples at 48 h after elicitation. Representative pictures of wild-type (WT) mice and CX3CR1^−/−^ mice (*n* = 5, respectively). Original magnification ×100; scale bar = 200 μm. (**B**) The numbers of neutrophils, F4/80^+^ cells, CD3^+^ cells, and CD8^+^ cells per high power field in the skin samples of wild-type mice and CX3CR1^−/−^ mice at 48 h after elicitation. All values represent the mean ± SEM. *n* = 10. * *p* < 0.05 by Mann–Whitney U-test.

Skin samples were collected 48 h after DNFB challenge. Consistent with the decrease in ear swelling, dermal edema was attenuated in the ear of CX3CR1^−/−^ mice compared to wild-type mice (Figure 2A). In addition, CX3CR1^−/−^ mice had significantly fewer neutrophils infiltrating into the dermis than wild-type mice (Figure 2B). Macrophage and T cell numbers were also assessed in skin tissue sections stained with anti-F4/80 antibody, anti-CD3 antibody, and anti-CD8 antibody. There were no statistically significant differences in the numbers of T cells and macrophages in the dermis between CX3CR1^−/−^ mice and wild-type mice (Figure 2B), suggesting that CX3CR1 deficiency does not affect the migration of T cells and macrophages in CHS response induced by DNFB. Thus, CX3CR1 deficiency decreased infiltration of neutrophils, followed by attenuated dermal edema in DNFB-induced CHS response.

### 2.3. TNF-α and IL-6 Expression Was Downregulated in CX3CR1^−/−^ Mice in DNFB-Induced CHS Response

**Figure 3:**
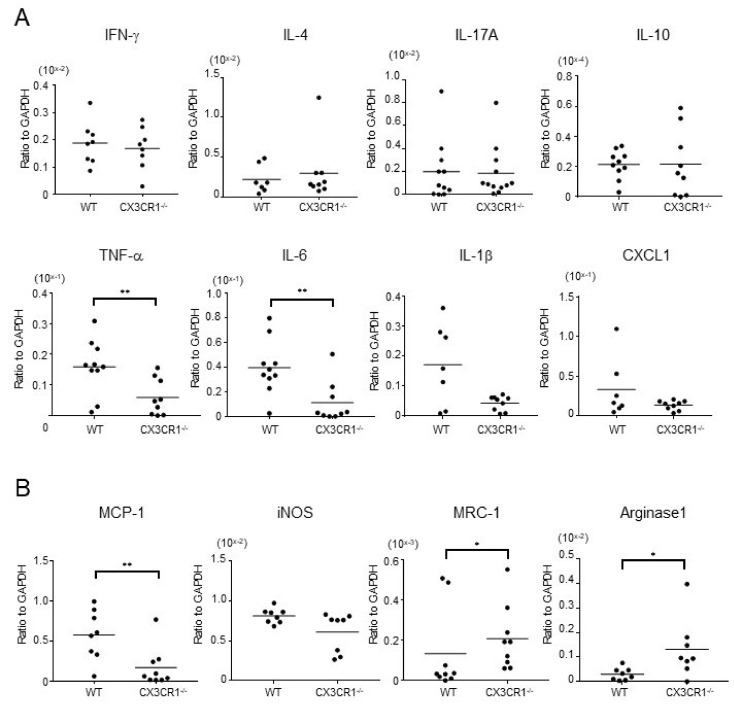
Expression of cytokines and chemokines produced by M1 macrophages is decreased and M2 macrophage marker expression is increased in the lesional skin of CX3CR1^−/−^ mice. (**A**) mRNA expression of interferon (IFN)-γ, interleukin (IL)-4, IL-17A, IL-10, tumor necrosis factor (TNF)-α, IL-6, IL-1β, and CXCL1 in the ears from wild-type mice and CX3CR1^−/−^ mice at 48 h after DNFB challenge. Horizontal bars indicate mean values for each group of mice. *n* = 7–12. ** *p* < 0.01 by Mann–Whitney U-test. (**B**) mRNA expression of monocyte chemoattractant protein (MCP)-1, inducible nitric oxide synthase (iNOS), mannose receptor C type 1 (MRC-1), and arginase-1 in the ears from wild-type mice and CX3CR1^−/−^ mice at 48 h after DNFB challenge. Horizontal bars indicate mean values for each group of mice. *n* = 8–9. * *p* < 0.05, ** *p* < 0.01 by Mann–Whitney U-test.

We next examined mRNA expression of inflammatory cytokines in the ear 48 h after the DNFB challenge. There were no significant differences in the expression levels of interferon (IFN)-γ, a representative Th1 cytokine, IL-4, a representative Th2 cytokine, and IL-17A, a representative Th17 cytokine, between CX3CR1^−/−^ mice and wild-type mice (Figure 3A). In addition, IL-10 mRNA expression levels in CX3CR1^−/−^ mice were also comparable to those in wild-type mice (Figure 3A). On the other hand, expression of inflammatory cytokines such as tumor necrosis factor (TNF)-α and IL-6 were significantly lower in CX3CR1^−/−^ mice than wild-type mice (*p* < 0.01 and *p* < 0.01, respectively; Figure 3A). IL-1β mRNA expression levels also tended to be attenuated in CX3CR1^−/−^ mice (Figure 3A). In addition, CXCL1, a major neutrophil chemoattractant, also tended to be decreased in CX3CR1^−/−^ mice (Figure 3A). Thus, decreased CHS response by DNFB challenge was accompanied by the reduced TNF-α and IL-6 expression in CX3CR1^−/−^ mice.

### 2.4. M1 Macrophage Marker Expression Was Downregulated and M2 Macrophage Marker Expression Was Upregulated in CX3CR1^−/−^ Mice

Macrophages are one of the main sources of inflammatory cytokines, including TNF-α, IL-6, and IL-1β. Moreover, macrophages also produce various chemoattractants for neutrophils including CXCL1. CX3CR1 is expressed on macrophages and is associated with their function [15]. Therefore, we further investigated mRNA expression levels of various macrophage markers in the dermis of DNFB-induced CHS response. The expression levels of monocyte chemoattractant protein (MCP)-1 were significantly decreased in CX3CR1^−/−^ mice compared with wild-type mice (*p* < 0.01, Figure 3B). The levels of inducible nitric oxide synthase (iNOS) were also decreased in some CX3CR1^−/−^ mice, although the difference did not reach statistical significance (Figure 3B). MCP-1 and iNOS were reported to be produced by M1 macrophages [18]. In contrast, CX3CR1^−/−^ mice revealed significantly higher expression of mannose receptor C type 1 (MRC-1) and arginase-1, both of which are M2 macrophage markers [19] (*p* < 0.05, respectively; Figure 3B). Thus, M1 macrophage marker expression was decreased in the ear of DNFB-induced CHS in CX3CR1^−/−^ mice, while M2 macrophage marker expression was increased.

### 2.5. TNF-α and IL-6 Expression Was Decreased and Arginase-1 Expression Was Increased in Macrophages from CX3CR1^−/−^ Mice

**Figure 4:**
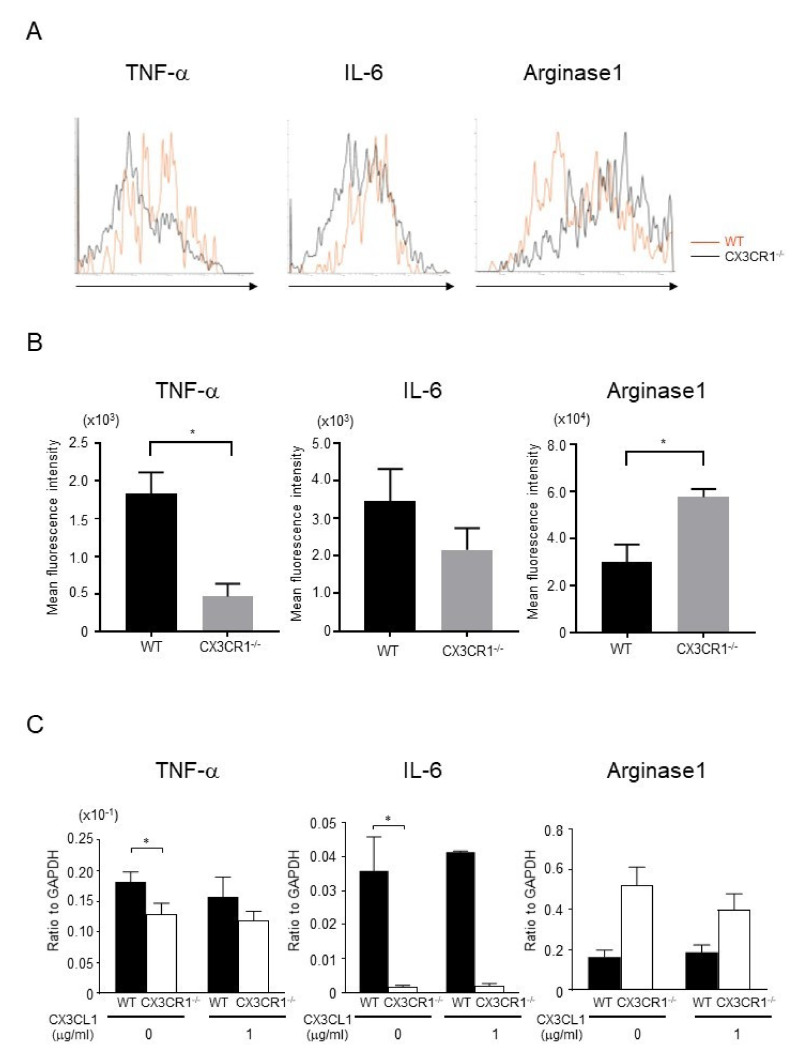
TNF-α and IL-6 expression was decreased and arginase-1 expression was increased in macrophages from CX3CR1^−/−^ mice. (**A**) Cells were collected from the skin at 48 h from DNFB challenge. TNF-α, IL-6, and arginase-1 expression from F4/80^+^ cells was assessed by intracellular flow cytometric analysis. Representative histograms comparing WT mice and CX3CR1^−/−^ mice. (**B**) Mean fluorescence intensities of TNF-α, IL-6, and arginase-1. All values represent the mean ± SEM. *n* = 3–4. Results are representative of two experiments with similar findings. * *p* < 0.05 by Mann–Whitney U-test. (**C**) Peritoneal macrophages from wild-type mice and CX3CR1^−/−^ mice were cultured with soluble CX3CL1 (0, 1 μg/mL) for 24 h. TNF-α, IL-6, and arginase-1 mRNA expression levels were measured by real-time PCR. All values represent the mean ± SEM. *n* = 4. Results are representative of three experiments with similar findings. * *p* < 0.05 by Mann–Whitney U-test.

To assess cytokine expression in macrophages, we collected cells from the skin after DNFB challenge and performed intracellular flow cytometric analysis for TNF-α, IL-6, and arginase-1. TNF-α expression in F4/80^+^ cells was significantly decreased in CX3CR1^−/−^ mice compared to wild-type mice, whereas arginase-1 expression was significantly increased in CX3CR1^−/−^ mice (Figure 4A,B). IL-6 expression tended to decrease in CX3CR1^−/−^ mice, although there was no statistical significance (Figure 4A,B). As the number of macrophages in the skin was very small, we next focused on peritoneal macrophages. We isolated peritoneal macrophages from untreated wild-type mice and CX3CR1^−/−^ mice and stimulated them with soluble CX3CL1 (1 μg/mL) for 24 h. Expression levels of TNF-α and IL-6 mRNA by unstimulated peritoneal macrophages from CX3CR1^−/−^ mice were decreased (Figure 4C), consistent with the previous report [15]. Moreover, arginase-1 mRNA expression was increased in unstimulated peritoneal macrophages from CX3CR1^−/−^ mice (Figure 4C). However, soluble CX3CL1 stimulation did not affect the expression levels of TNF-α, IL-6, and arginase-1 (Figure 4C). Thus, in CX3CR1^−/−^ mice, macrophages had less capacity to produce inflammatory cytokines and more capacity to produce arginse-1.

### 2.6. Depletion of Macrophages Ameliorated DNFB-Induced CHS Response in Wild-Type Mice to the Same Extent as CX3CR1^−/−^ Mice

**Figure 5:**
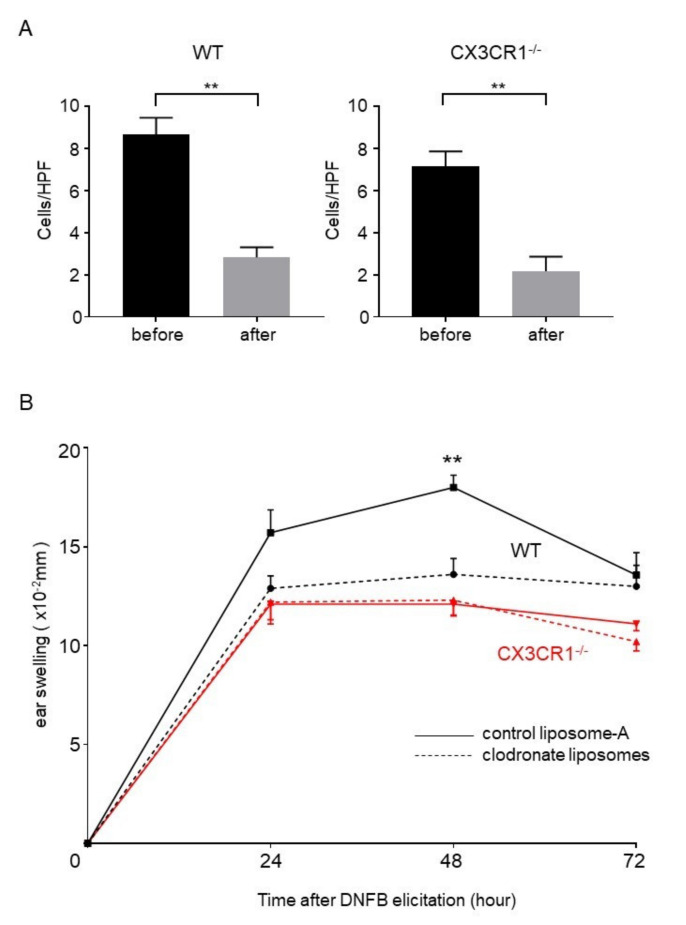
Depletion of dermal macrophages in wild-type mice attenuates CHS to the same extent as CX3CR1^−/−^ mice. (**A**) The number of F4/80^+^ cells per high power field in the skin samples of wild-type mice and CX3CR1^−/−^ mice before and after injection of clodronate liposomes. All values represent the mean ± SEM. *n* = 6. ** *p* < 0.01 by Mann–Whitney U-test. (**B**) Challenge by DNFB was performed one day after intradermal injection of clodronate liposomes or control liposomes into wild-type mice or CX3CR1^−/−^ mice. Ear swelling was measured before elicitation and at 24, 48, and 72 h after elicitation and the changes in ear thickness from baseline measurement were presented. All values represent the mean ± SEM. *n* = 7–10. ** *p* < 0.01 by Kruskal–Wallis test and Steel–Dwass test.

Based on the results above, we hypothesized that decreased inflammatory cytokine expression and increased arginase-1 expression in macrophages could cause attenuated CHS response in CX3CR1^−/−^ mice. It has been known that macrophage depletion by clodronate liposomes before elicitation but not sensitization significantly inhibits CHS response [20]. To directly evaluate the involvement of macrophages from CX3CR1^−/−^ mice in decreased CHS response, clodronate liposomes were injected into elicitation sites 24 h before elicitation. We first confirmed that injection of clodronate liposomes decreased the number of F4/80^+^ cells in the dermis both in wild-type mice and CX3CR1^−/−^ mice (Figure 5A). Ear swelling of wild-type mice was significantly decreased by depletion of dermal macrophages to similar levels of CX3CR1^−/−^ mice (Figure 5B). In contrast, depletion of dermal macrophages did not affect the ear swelling of CX3CR1^−/−^ mice after DNFB challenge (Figure 5). These results suggest that macrophages play an important role in DNFB-induced CHS response and that macrophages with downregulated inflammatory cytokines and upregulated arginase-1 in CX3CR1^−/−^ mice are involved in attenuation of DNFB-induced CHS response.

## 3. Discussion

In this study, we first showed that CHS response by DNFB was attenuated in CX3CR1^−/−^ mice compared with wild-type mice. Although CHS is mainly mediated by Th1 cells and CD8^+^ cytotoxic T cells and suppressed by regulatory T cells in elicitation phase, the infiltration of CD3^+^ T cells in the dermis was not affected in CX3CR1^−/−^ mice. In addition, there were no significant differences in IFN-γ, IL-4, IL-17A, and IL-10 expression levels between CX3CR1^−/−^ mice and wild-type mice. By contrast, in the skin from CX3CR1^−/−^ mice, expression levels of inflammatory cytokines, such as TNF-α and IL-6, were decreased. In addition, expression of CXCL1, a major chemoattractant for neutrophils, and the number of infiltrating neutrophils were also decreased. Neutrophils barely expressed CX3CL1 [21,22]. Moreover, although CX3CR1 is expressed on neutrophils [9,23], soluble CX3CL1 is not the major chemoattractant for neutrophils [24,25]. Based on these reports, the decreased neutrophils in CX3CR1^−/−^ mice might be induced by downregulation of CXCL1 but not CX3CR1 deficiency in neutrophils. Consistently, CX3CR1 does not regulate accumulation of neutrophils in wounded skin [22]. Comprehensively, our results suggest that downregulation of inflammatory cytokines and neutrophil-chemoattractants can cause the attenuation of DNFB-induced CHS response.

We previously reported that imiquimod-induced psoriasis-like skin inflammation was attenuated in CX3CR1^−/−^ mice through decrease in M1 macrophages but not total macrophages [15]. Similar to the report, the number of F4/80^+^ total macrophages in the dermis was not decreased in CHS responses of CX3CR1^−/−^ mice, suggesting that other chemokine receptors, such as CCR2, may compensate for loss of CX3CR1 in terms of macrophage migration. Consistent with the hypothesis, interestingly, CX3CL1 mRNA expression in the skin was not increased at 48 h after DNFB challenge (data not shown). Then, we focused on the function of macrophages as the cause of attenuated CHS response in CX3CR1^−/−^ mice. Macrophages are classified into M1 macrophages and M2 macrophages. M1 macrophages, which produce inflammatory cytokines such as TNF-α, IL-6, IL-1β, IL-12, and IL-23, and reactive oxygen species, mediate not only antimicrobial and antitumoral activity but also tissue inflammation [26,27,28]. On the other hand, M2 macrophages antagonized the inflammatory responses by M1 macrophages through producing suppressive cytokines such as IL-10, TGF-β, and arginase-1 [18,27]. In this study, expression of MCP-1, one of the M1 macrophage markers [18], was significantly reduced in the skin of CHS in CX3CR1^−/−^ mice compared to wild-type mice. In addition, TNF-α and IL-6 expression were downregulated in unstimulated peritoneal macrophages from CX3CR1^−/−^ mice, consistent with our previous report on imiquimod-induced psoriasis-like skin inflammation model in CX3CR1^−/−^ mice [15]. Moreover, we found that expression of MRC-1 and arginase-1, which are M2 macrophage markers [19], was significantly higher in CX3CR1^-/-^ mice and that arginase-1 expression was upregulated in CX3CR1-deficient peritoneal macrophages without any stimulation. These results suggest that not only a decrease in M1 macrophages but also skewed macrophage polarization towards M2 phenotype occurred in mice with genetic loss of CX3CR1. Consistently, CX3CR1^−/−^ mice show smaller infarcts in the model of middle cerebral artery occlusion accompanied by the increase in M2 polarization markers [29]. Recently, Lee et al. reported that F4/80^high^ hepatic macrophages in CX3CR1-deficient mice showed reduced expression of IL-1β and TNF-α and elevated expression of arginase-1, resulting in amelioration of alcoholic liver injury [30]. They also found that IL-1β and TNF-α expression were decreased and IL-10 expression was increased in monocytes co-cultured with CX3CL1-depleted endothelial cells compared with those co-cultured with control endothelial cells, suggesting that the interaction between CX3CL1 and CX3CR1 is associated with inflammatory phenotype of macrophages. In this report, we showed that soluble CX3CL1 failed to affect cytokine expression in peritoneal macrophages. Comprehensively, the lack of stimulation from membrane-bound CX3CL1 but not soluble CX3CL1 in CX3CR1^−/−^ mice might cause the skewed polarization towards M2 phenotype.

Inconsistent with our results, Nakagomi et al. reported that MMP-12 produced by intradermally-injected bone marrow-derived mannose receptor-positive M2 macrophages into naïve mice exacerbated CHS symptoms [31]. They also showed that CHS was ameliorated in MMP-12^−/−^ mice, indicating that MMP-12 has the capacity to aggravate CHS responses. To solve the discrepancy between the previous report and our result, we examined MMP-12 mRNA expression in the skin after DNFB challenge and peritoneal macrophages. Interestingly, MMP-12 expression in CX3CR1^−/−^ mice was not increased both in the skin and macrophages (data not shown). According to the result, not only skewed M2 polarization but also characteristic changes in M2 macrophages might occur in CX3CR1^−/−^ mice.

Although T cells are the key mediators during CHS elicitation, emerging evidence recently suggests that macrophages also play an important role in CHS challenge [18]. For example, arginase-1 deficiency in LysM^+^ cells including monocytes and macrophages resulted in exacerbation of DNFB-induced CHS response through upregulated iNOS expression [18]. Dermal macrophages play an important role in forming dendritic cell clusters with effector T cells in dermal perivascular areas via IL-1 receptor signaling in DNFB-induced CHS response [32]. Moreover, glucocorticoids can ameliorate CHS in mice with T-cell-specific glucocorticoid receptor deficiency but fail to suppress CHS in macrophage-specific glucocorticoid-receptor-deficient mice [3]. Consistently, we found that dermal macrophage depletion in wild-type mice before elicitation phase suppressed DNFB-induced CHS response to a comparable level of CX3CR1^−/−^ mice and that depletion of macrophages did not affect the CHS response in CX3CR1^−/−^ mice. Taken together, decreased inflammatory cytokine expression and increased arginase-1 expression in macrophages could cause the attenuation of DNFB-induced CHS response in CX3CR1^−/−^ mice.

In conclusion, our results support the notion that macrophages also play an important role in contact dermatitis and that CX3CR1 deficiency might affect the polarization and function of macrophages. Targeting macrophages or CX3CR1 could be a new therapeutic strategy for the treatment of allergic contact dermatitis.

## 4. Materials and Methods

### 4.1. Mice

CX3CR1^−/−^ mice with C57BL/6 background had been established by gene targeting as described elsewhere [33]. CX3CR1^−/−^ mice and C57BL/6 mice were obtained from the Jackson Laboratory (Bar Harbor, ME, USA). All mice used were 8 to 12 weeks old. They were healthy, fertile, and did not display evidence of infection or disease. All animal experiments were approved by the Animal Research Committee of the University of Tokyo (Tokyo, Japan) on 15 February 2018 (Project code: P17-102).

### 4.2. Sensitization and Elicitation of CHS

CHS responses were induced with DNFB (Sigma-Aldrich, St Louis, MO, USA), as previously described [34]. Briefly, a volume of 50 µL of 0.5% DNFB, in acetone and olive oil (4:1), was painted onto shaved abdominal skin on day 0 and 1. CHS was elicited by applying 0.5% DNFB on the right ear on day 6. For all CHS experiments, baseline ear thickness was determined with a spring-loaded caliper. Ear swelling was measured before elicitation and at 24, 48, and 72 h after elicitation. The changes in ear thickness from baseline measurement were computed. In some experiments, mice were subcutaneously injected into their right ear with 20 μL of chlodronate liposomes or control liposomes (Formu Max, Sunnyvale, CA, USA) to deplete cutaneous macrophages 24 h before the elicitation as previously described [35].

### 4.3. Histological and Immunohistochemical Analysis

Skin samples were collected from wild-type and CX3CR1^−/−^ mice ears at 48 h after elicitation and assessed for tissue inflammation and number of infiltrating neutrophils, macrophages, and T cells. A central strip of the ear was fixed in 10% formalin and then paraffin embedded. Sections, 6 µm thick, were stained using hematoxylin and eosin for evaluation of inflammatory changes and neutrophils and anti-mouse F4/80 Ab (Serotec, Oxford, UK) for evaluation of macrophages. Sections were incubated with Histofine Simple Stain mouse MAX-PO (Nichirei Biosciences, Tokyo, Japan; 30 min at room temperature). For evaluation of T cells, frozen tissue sections of skin biopsies were acetone-fixed and then incubated with 5% normal rabbit serum in phosphate-buffered saline (10 min at 37 °C) to block nonspecific staining. Sections were then incubated with goat polyclonal Abs specific for CD3 (Santa Cruz Biotechnology, Dallas, TX, USA), CD8 (Santa Cruz Biotechnology), or Goat IgG (Vector Laboratories, Burlingame, CA, USA), followed by ABC staining (Vector Laboratories, Burlingame, CA, USA). Diaminobenzidine was used for visualizing the staining, and counterstaining with Mayer hematoxylin was performed, according to the manufacturers’ instructions. Infiltration of dermal neutrophils, F4/80^+^ cells (macrophages), CD3^+^ T cells, and CD8^+^ T cells was evaluated by averaging the number of them counted in 10 random grids under high-magnification (×400) power fields of a light microscope. Each section was examined independently by three investigators in a blinded manner and the mean was used for analysis.

### 4.4. RNA Isolation and Quantitative Reverse Transcription-PCR

RNA was obtained from the ears with RNeasy Fibrous Tissue Mini Kit (QIAGEN, Valencia, CA, USA) or peritoneal macrophages with Trizol Reagent (Invitrogen, Carlsbad, CA, USA). Complementary DNA was synthesized using ReverTra Ace^®^ qPCR RT Master Mix (TOYOBO, Osaka, Japan). Expression levels of IFN-γ, IL-4, IL-17A, TNF-α, IL-6, IL-1β, IL-10, CXCL1, MCP-1, iNOS, MRC-1, and arginase-1 mRNA were analyzed by a real-time PCR quantification method with THUNDERBIRD SYBR qPCR Mix (TOYOBO, Osaka, Japan) on an ABI Prism 7000 sequence detector (Applied Biosystems, Foster City, CA, USA). Glyceraldehyde-3-phosphate dehydrogenase (GAPDH) was used to normalize the mRNA. All samples were analyzed in parallel for GAPDH gene expression as an internal control. The relative expression levels of each gene were determined by the 2^−ΔΔCT^ method.

Primers used were as follows: murine IFN-γ forward, 5′-AGC AAC AGC AAG GCG AAA A-3′ and reverse, 5′-CTG GAC CTG TGG GTT GTT GA-3′; murine IL-4 forward, 5′-ACG GAG ATG GAT GTG CCA AAC GTC-3′ and reverse, 5′-CGA GTA ATC CAT TTG CAT GAT GC-3′; murine IL-17A forward, 5′-CAG CAG CGA TCA TCC CTC AAA G-3′ and reverse, 5′-CAG GAC CAG GAT CTC TTG CTG-3′; murine TNF-α forward, 5′-CCA CCA CGC TCT TCT GTC TAC-3′ and reverse, 5′-AGG GTC TGG GCC ATA GAA CT-3′; murine IL-6 forward, 5′-AGT TGC CTT CTT GGG ACT GA-3′, and reverse, 5′-TCC ACG ATT TCC CAG AGA AC-3′; murine IL-1β forward, 5′-ACC TGT CCT GTG TAA TGA AAG-3′ and reverse, 5′- GCT TGT GCT CTG CTT GTG-3′; murine IL-10 forward, 5′-TTT GAA TTC CCT GGG TGA GAA-3′ and reverse, 5′-ACA GGG GAG AAA TCG ATG ACA-3′; murine CXCL1 forward, 5′-GGC TGG GAT TCA CCT CAA GAA C-3′ and reverse, 5′-TGT GGC TAT GAC TTC GGT TTG G-3′; murine MCP-1 forward, 5′-CTG GAT CGG AAC CAA ATG AG-3′ and reverse, 5′-CGG GTC AAC TTC ACA TTC AA-3′; murine iNOS forward, 5′-CGA AAC GCT TCA CTT CCA A-3′ and reverse, 5′-TGA GCC TAT ATT GCT GTG GCT-3′; murine MRC-1 forward 5′-GCA AAT GGA GCC GTC TGT GC-3′ and reverse, 5′-CTC GTG GAT CTC CGT GAC AC-3′; murine arginase-1 forward, 5′-ATG GAA GAG ACC TTC AGC TAC-3′ and reverse, 5′-GCT GTC TTC CCA AGA GTT GGG-3′; murine GAPDH forward, 5′-CGT GTT CCT ACC CCC AAT GT-3′ and reverse, 5′- TGT CAT CAT ACT GGC AGG TTT CT-3′.

### 4.5. Intracellular Flow Cytometric Analysis of Dermal Macrophages

Excised ear skin was cut into small pieces with 400 µL Minimum Essential Media. After 60 mg collagenase type2 (Worthington, Lakewood, NJ, USA) and 1.2 mg CaCl_2_ were added into 30 mL Minimum Essential Media, 10 mL solution was added into each cut skin with shaking at 70 rpm for 90 min at 37 °C. The solution was filtered using a 45 µm cell strainer, and centrifuged at 1800 rpm for 10 min at 4 °C. The cells in the solution were stained with PE-Cyanine7-conjugated anti-mouse F4/80 Ab (eBioscience, San Jose, CA, USA). After surface staining, the cells were permeabilized using fixation/permeabilization buffer (eBioscience) and APC-conjugated anti-mouse TNF-α Ab (eBioscience), PE-conjugated anti-mouse IL-6 Ab (eBioscience), and PE-conjugated anti-arginase-1 Ab (eBioscience) were added. Cells were washed and analyzed on a FACScan flow cytometer (BD PharMingen, San Jose, CA, USA).

### 4.6. Isolation of Peritoneal Macrophage

Mouse peritoneal macrophages were harvested by washing the peritoneal cavity with ice-cold 10 mL RPMI 1640 containing 10% phosphate-buffered saline and incubated for 2 h. After gentle agitation the cells were washed three times with RPMI 1640 to remove nonadherent cells before further manipulation. We confirmed that most infiltrating cells in peritoneal lavage were stained positive with FITC-conjugated anti-mouse F4/80 Ab (eBioscience, San Jose, CA, USA) by a FACScan flow cytometer (BD PharMingen, San Jose, CA, USA). 1 × 10^6^ cells of peritoneal lavage from CX3CR1^−/−^ mice and wild-type mice were cultured with or without CX3CL1 (1 μg/mL; R&D systems, Minneapolis, MN, USA) for 24 h.

### 4.7. Statistics

Statistical analysis was performed using Prism Version 7 software (GraphPad, San Diego, CA, USA). All data were shown as mean values ± SEM. Statistical analysis between 2 groups was performed using Mann–Whitney U-test. The Kruskal–Wallis test and Steel–Dwass test were used for multiple comparisons. *p*-values of < 0.05 were considered statistically significant.
